# Heart Failure and Mechanical Circulatory Assist Devices

**DOI:** 10.5539/gjhs.v5n5p11

**Published:** 2013-05-14

**Authors:** Eluisa La Franca, Rosanna Iacona, Laura Ajello, Angela Sansone, Marco Caruso, Pasquale Assennato

**Affiliations:** 1 Division of Cardiology, University Hospital “P. Giaccone” of the University of Palermo, Palermo, Italy

**Keywords:** mechanical circulatory support, VAD, INTERMACS, heart failure, destination therapy, cardiac transplantation

## Abstract

During the last 20 years, the management of heart failure has significantly improved by means of new pharmacotherapies, more timely invasive treatments and device assisted therapies. Indeed, advances in mechanical support, namely with the development of more efficient left ventricular assist devices (LVAD), and the total artificial heart have reduced mortality and morbidity in patients with end-stage heart failure awaiting for transplantation. However, the transplant cannot be the only solution, due to an insufficient number of available donors, but also because of the high number of patients who are not candidates for severe comorbidities or advanced age. New perspectives are emerging in which the VAD is no longer conceived only as a “Bridge to Transplant”, but is now seen as a destination therapy. In this review, the main VAD classification, current basic indications, functioning modalities, main limitations of surgical VAD and the total artificial heart development are described.

## 1. Introduction

Heart failure (HF) is one of the main causes of death in Western Countries. Its prevalence (1-2 % in the general population) increases exponentially with age. Over the next 3 decades, the population of individuals aged ≥65 years with HF is expected to be more than double, so we can assume that the number of individuals at risk of HF will increase dramatically in the near future (Consensus conference on the management of heart failure, 2006; Ansari & Massie, 2003).

### 1.1 Rationale for the Use of Ventricular Assist Devices

Because of the impossibility of carrying out a conventional surgical procedure in refractory HF, the only therapeutical solution in this case is that of a cardiac transplantation or, if the patient is hemodynamically unstable, the implantation of a ventricular assist device (VAD) as a bridge to transplant support (Hosenpud, Bennett, Keck, Boucek, & Novick, 2000). This term includes the biventricular assist devices (which actually replace the patient's heart) and those assist devices projected to support the pump function of one (RVAD, LVAD) or both ventricles (BVAD) (Colombo, 2006). The development of VAD led to the realization of several models which differ not only for the kind of technologies used and the implant position but also for the kind of flow they generate (pulsatile or continuous). Initially conceived as a “bridge to transplantation” support, only later has the use of VAD been extended to patients who are not candidates for heart transplant (“destination therapy”).

Nowadays, the main objectives of a mechanical circulatory support program is to hemodynamically stabilize patients with acute or chronic HF and to preserve the organ function in order not to preclude subsequent therapeutical options. Patients with end stage HF usually have symptoms at rest and a significant impairment of quality of life and are encumbered by a 1-year mortality of approximately 50% (Redfield, 2002; Stewart, MacIntyre, Hole, Capewell, & McMurray, 2001). These patients are generally candidates for cardiac transplantation. The cardiac transplantation is actually the “gold standard” treatment for the end stage HF but only 200-250 heart transplantations are performed each year (Redfield, 2002; Hunt, 2006) with a significant increase of patients on the waiting list. The result is the increase in the average waiting time (currently more than 2 years) that, together with the extension of the indications for transplantation, result in a high risk for patients experiencing an acute, often irreversible, hemodynamic deterioration. Since 2002 the mechanical circulatory support program has been approved from Food and Drug Administration also as a replacement therapy (destination therapy) in patients with indications but who are not candidates for cardiac transplantation.

## 2. Classification of the Devices

The term VAD means a mechanical circulatory support that is temporarily or permanently able to replace cardiac function in response to the needs of the organism. In other words it substitutes or supports a heart that is not able to perform its pump function. VADs are designed to assist either the right (RVAD) or left (LVAD) ventricle, or both at once (BiVAD); VADs need to be clearly distinguished from artificial hearts, which are designed to take over cardiac function completely.

The pumps used in VADs can be divided into two main categories: pulsatile pumps, that mimic the natural pulsing action of the heart, and continuous flow pumps. On the basis of the implant location, VADs can be distinguished in extracorporeal, paracorporeal and intracorporeal devices.

### 2.1 Pulsatile Flow Pumps

In these devices, the inflow and outflow cannulas are equipped with artificial valves to allow unidirectional flow; the artificial ventricular pump receives blood from the cardiac chambers through the inflow cannula, that can be placed in the left or right atrium or in the ventricular apex; the outflow cannula returns blood to either the aorta (in a left ventricular assist device, LVAD) or pulmonary artery (in a right ventricular assist device, RVAD). The pump is connected to a pneumatic driver: the driver provides alternating pneumatic pressure and vacuum in a pulse-like fashion for ejection and filling. In general, the filling of the artificial ventricle depends on the atrial filling pressure and on the entity of the suction. Output of the pump depends on intravascular volume status and downstream vascular resistances.

The first generation devices were extracorporeal: they were located externally to the patient and mounted on a pole. They were designed for short-term use and burdened with a high rate of complications; in addition, the patient was compelled to stay in bed. An example is the system Abiomed BVS 5000.

Subsequently, paracorporeal VADs (PVADs) have been developed. There are several advantages in the design of PVADs: the paracorporeal location allows for right, left or biventricular support and ease of exchange in the event of device malfunction, thrombosis or infection; what is more, a portable driver is available so that the patient can be discharged from hospital. These systems, still in use today, can act as a “bridge to transplant” or “bridge to recovery” support. The most used devices of this category are Berlin Heart Excor and Thoratec.

A significant advance is represented by intracorporeal systems (IVADs): these are implantable pulsatile pumps, pneumatically or electrically driven. These devices can support only the left ventricle and are equipped with unidirectional valves. Because of their dimensions, the implant is abdominal, intra- or extraperitoneal. The pump is connected to the cardiac chambers through inflow and outflow cannulas. A percutaneous line connects the device to an external driver. Nowadays, the most used models of this category are Heartmate I XVE and Novacor. The main disadvantages are represented by their size (which makes smaller patients ineligible for the implant), the presence of longer lines of connection (which may be a source of infections) and the high rate of thromboembolic events (Pae, 2007; Rose, 2001).

### 2.2 Continuous Flow Pumps

There has been a trend in the evolution in the VADs from extracorporeal pulsatile to implantable pulsatile to implantable continuous flow. Newer generation LVADs have been developed with continuous flow, rotary pump technology and are smaller and quieter than older pulsatile volume displacement LVADs. Continuous flow VADs normally use either centrifugal pumps or an axial flow pump: unlike the other pumps, which completely unload the ventricle, many patients with an axial flow pump maintain a pulsatile pattern of blood flow. The partial dependence of these devices from gradients of pump (pressure difference between inflow and outflow) in terms of induced flows, suggests using them when a minimum residual left ventricular function is guaranteed, stressing that they are only LVAD.

Obviously, the higher the residue pressure produced from the ventricle, the lower the gradients of pump.

Their small size allows implantation into smaller patients (with possible developments as pediatric assist device in the near future), makes placement easier and makes them extremely interesting as a destination therapy. This category of devices can be classified on the basis of the implant position: on one side the Impella (extracorporeal) system and the extracorporeal and paracorporeal centrifugal pumps, on the other the most used intracorporeal axial pumps such as Berlin Heart Incor, De Bakey, Jarvik 2000, Heartmate II.

### 2.3 Total Artificial Heart (TAH)

On April 4, 1969, Liotta and Cooley replaced a dying man's heart with a mechanical heart inside the chest at The Texas Heart Institute in Houston as a bridge for a transplantation. The patient woke up and recovered well. After 64 hours, the pneumatic-powered artificial heart was removed and replaced by a donor heart. The TAH is the most developed implantable pump. It replaces the patient's heart and the ventricular function. It is used as a bridge for a transplantation. The patient woke up and recovered well. After 64 hours, the pneumatic-powered artificial heart was removed and replaced by a donor heart. The TAH is the most developed implantable pump. It replaces the patient's heart and the ventricular function. It is used as a bridge to transplantation support. It's important to ensure a balance between the stroke volume of both ventricles and adjust the flow to the physiological needs of the organism. Among TAH, two models are currently FDA approved: AbiomedAbioCor Total Artificial Heart and Jarvik 7-CardioWest. Their clinical use is far from being widespread, but important developments are expected in the future. The only situations in which their application is indicated are represented by patients waiting for cardiac transplantation in which no other kind of assistance is possible.

## 3. Strategies of Use and Implant of VADs and Clinical Implications

Depending on the use, the mechanical circulatory support can be distinguished in temporary, mid-term and long-term (Table 1).

**Table 1:** Indications for ventricular assist device

Indications for VAD	Rationale
“Bridge to Transplantation”:	Mechanical circulatory support in patients, who are eligible for cardiac transplantation, in critical hemodynamic condition despite maximal inotropic support, in order to “gain time“ and find a compatible heart.
“Bridge to Recovery”:	Mechanical circulatory support aimed at the recovery of cardiac function. The volume and pressure unloading of the left ventricle, the simultaneous restoration of systemic blood pressure and the normalization of the neurohormonal and cytokine milieu are responsible for the reverse remodeling so as to allow, in some cases, the device removal.
“Destination Therapy”:	Long-term mechanical circulatory support in patients not eligible for heart transplantation.
“Bridge to Candidacy”:	Mid- or long-term mechanical circulatory support for some marginal recipients with potentially reversible contraindications to transplant.
“Bridge to Bridge”:	Short-term mechanical circulatory support in postcardiotomy patients bridging them to a long-term implantable VAD.

**Duration of treatment**	**Rationale**
Temporary < 1 month	Intra aortic balloon pump, Impella, centrifugal pumps, extracorporeal VAD
Short term	mono or biventricular paracorporeal VAD
Brige < 1 year	Total artificial heart, implantable VAD
Long term > 1 year	Implantable VAD

**VADs**: ventricular assist device

To date, there are no consensus guidelines for VAD implantation (Boyle, 2009; Messori, Trippoli, Bonacchi, & Sani, 2009; Park, 2005). However, an appropriate selection of candidates is the key to success of implantation (Lietz, 2007; Miller, 2003) and there is strong evidence derived from clinical trials that can be used as guidelines. Mechanical circulatory support is almost always used in case of cardiogenic shock or other situations that are potentially reversible (bridge to recovery) (Bonacchi, Maiani, Harmelin, & Sani, 2009; Holman, Bourge & Kirklin, 1991; Mancini et al., 1998) or in candidates who are either “too sick” to wait for a donor to be identified because of severe acute, or acute-on-chronic HF, or have contraindications to transplantation, which are deemed to be transient (bridge to transplantation) (Bonacchi et al., 2009; Frazier et al., 1995; Miller et al., 2007). In these situations, a mono- or biventricular assist device could be necessaryand the outcomes depend on the complications related to the mechanical support and the clinical condition of the patient before the implant. The encouraging results obtained with pulsatile, electrically driven VADs (Haft et al., 2007; Potapov et al., 2006; Westaby, 2004; Westaby, Frazier, & Banning, 2006) permit to consider their use as destination therapy or long term therapy in order to allow the patient to resume normal activities (Table 1). There is also the opportunity, to date demonstrated in a selected category of patients, to obtain a reverse remodeling through the minimization of myocardial work and the administration of the appropriate therapy so that, once the heart function has been restored, it is possible to remove the device (Bonacchi et al., 2009; Maybaum et al., 2007).

Currently, VADs seem to be significantly underused because of their elevated costs, not considering their therapeutical and economic advantages (Bonacchi, Massetti, & Sani, 2010). Strong evidence supporting their benefits is reported in several studies. In the REMATCH trial (Randomized Evaluation of Mechanical Assistance for the Treatment of Congestive Heart Failure) (Lietz et al., 2007), 129 patients with end-stage heart failure who were ineligible for cardiac transplantation were randomly assigned to receive a left ventricular assist device (68) or optimal medical management (61). All patients had symptoms of New York Heart Association (NYHA) class IV heart failure. The rates of survival after a year were 52 percent in the device group and 25 percent in the medical-therapy group (p = 0.002), and the rates after two years were 23 percent and 8 percent (p = 0.09), respectively. Similar results were obtained in the INTREPID trial (Investigation of Non-Transplant-Eligible Patients who are Inotrope Dependent Study) (Rogers et al., 2007). In this trial, fifty-five patients with NYHA functional class IV symptoms who failed weaning from inotropic support were offered a Novacor LVAD. Eighteen of these patients did not receive an LVAD owing to patient preference (n = 14) or unavailability of the device (n = 4). The LVAD-treated patients had superior survival rates at 6 months (46% vs. 22%; p = 0.03) and 12 months (27% vs. 11%; p = 0.02). Adverse event rates were higher in the OMT group. From these data, it is possible to assume the rationale for the use of VADs as destination therapy in elderly patients who are not candidates for transplantation and who are dependent on inotropic therapy.

The improvement in results related to the use of latest generation VADs leads to reconsider the clinical care pathways of patients in order to find better solutions. The relatively young patients waiting for transplantation should be subjected to mechanical assistance because otherwise they have a poor prognosis (Hunt, 2006; Boyle, 2009; Lee et al., 2003). Older patients (60-65 years) waiting for transplantation from long time, severely symptomatic, requiring frequent hospitalizations, with poor quality of life and poor prognosis at short-term (1-2 years) (Rogers et al., 2007), have little hope of being transplanted and in any case they would be with “marginal hearts”. Even in these cases there is a clear indication to the VAD system continuous-flow as definitive or however, long-term therapy. Similarly, patients who have an absolute or relative contraindication to transplantation (pulmonary hypertension, systemic diseases, etc.) have a well-defined indication to long-term therapy (Miller et al., 2007; Zimpfer et al., 2007). Despite all, however, the VAD implantation is seldom considered for patients with heart failure. Typically there is still the perception of VAD, based on experience of first-generation systems, characterized by mechanical dysfunctions, operational difficulties and frequent adverse events. The incomplete knowledge of the current results causes the possible implantation of VAD usually in terminal patients already in multiorgan failure (Lee et al., 2003; Stevenson & Couper, 2007). Rarely is given an indication for patiens in stable clinical condition, which would benefit from the use of a VAD. A more aggressive attitude would be supported by recent literature that suggests the extensive use of axial pumps assistance devices in patients belonging to class 4, 5 and 6 of INTERMACS (Stevenson & Couper, 2007). On the contrary, unfortunately, currently about 80% of VAD are used in patients classified as class 1 and 2 of INTERMACS. Another factor that explains the difficulty in the prevalence of VAD as destination therapy is that their use is often limited to transplant centers, while individuals not transplantable, but that could be candidates for the destination therapy, are not sent to these centers and therefore they are not evaluated in this light. To ensure the best care for these patients, a network for heart failure would be required with the aim of selecting and directing patients to appropriate structures able to deal with issues related to clinical status (Westaby et al., 2006).

## 4. Risk Factors and Complications

The most important factor for improving patient outcomes after LVAD is careful patient selection. In literature, people most likely to develop complications are those with: age > 60-70 years old; previous cardiosurgical interventions; liver disease; kidney failure with oliguria; infections; tumors; need for other mechanical support such as ventilation or ultrafiltration; cachexia as an index of chronic HF (Deng et al., 2001; Lund, Matthews, & Aaronson, 2010). The evaluation of right ventricular function has a crucial role in patient selection, since adequate LVAD function has to be warranted by adequate transpulmonary flow and right ventricular function. The presence of coronary artery disease is not a contraindication for VAD implantation: the improvement of the coronary perfusion guaranteed by the device should result in a reduction of myocardial ischemia. However, an appropriate antischemic therapy should be administered after the implantation, in order to protect the right ventricle. In addition, patients should not have contraindications to antiplatelet and anticoagulant therapy. The purely surgical problems include the presence of valvular heart disease or prosthetic valves, diseases of the thoracic aorta or intracardiac shunts.

Most of the complications related to the VAD are due to the therapies and to the technical aspects. The postoperative bleeding rate is rather high after VAD implantation, because of the aggressive anticoagulation regimen (Hoy, 2001; Slaughter et al., 2007). Patients with a VAD are frequently affected by bacterial infections that, if not well treated, can result in sepsis (Slaughter et al., 2007; Zierer et al., 2007). Thromboembolic events are a major complication of VAD implantation (Tsukui et al., 2007) and they are closely related to the activation of blood coagulation cascades due to blood-device interface and to turbulent flowin anyone of the conduits or in the device itself. Mechanical failure is less common due to technological advances that result in more reliable devices (Gambino et al., 2006; Pagani, Long, Dembitsky, Joyce, & Miller, 2006). Multiorgan failure (MOF) represents 30% of causes of death related to VADs. In many situations MOF is the end result of a long cascade of complications including sepsis, bleeding, and other events. In other casesit may be the result of multiorgan dysfunction that gets worse after the insult of surgery.

## 5. INTERMACS Report

The most used classification for HF, the NYHA and the ACC/AHA (Jessup et al., 2009), use to select patients who are suitable for VAD implantation if they are in NYHA functional class IV or in ACC/AHA stage D. Since 2006, The Interagency Registry For Mechanically Assisted Circulatory Support (INTERMACS), a National Heart Lung and Blood Institute–sponsored collaborative database, has been created and more than 6000 patients have been entered into the database with a primary device implant. The pre-implant INTERMACS levels reflect sub-sets of NYHA functional class IV and advanced class III. There are 7 different classes: level 1 refers to patients with critical cardiogenic shock; level 7 refers to those patients who are clinically stable but impaired with advanced HF (Table 2).

**Table 2:** INTERMACS levels adapted from US-Based Interagency Registry for Mechanical Circulatory Support – Profile description and Time frame for intervention (adapted from Stevenson & Couper, 2007)

INTERMACS Profile description	Time frame for intervention
**Level 1: Critical cardiogenic shock – “Crash and burn”**	Definitive intervention needed within hours
Patients with life-threatening hypotension despite rapidly escalating inotropic support, critical organ hypoperfusion, often confirmed by worsening acidosis and/or lactate levels.	
**Level 2: Progressive decline – “Sliding on inotropes”**	Definitive intervention needed within few days
Patients with declining function despite intravenous inotropic support; worsening renal function, nutritional depletion, inability to restore volume balance.	
**Level 3: Stable but inotrope dipendent – “Dependent stability”**	Definitive intervention elective over a period of weeks to few months
Patients who are stable on continuous intravenous inotropic support (or a temporary circulatory support device or both), but demonstrating repeated failure to wean from support due to recurrent symptomatic hypotension or renal dysfunction.	
**Level 4: Resting symptoms**	Definitive intervention elective over a period of weeks to few months
Patients can be stabilized close to normal volume status but experience daily symptoms of congestion at rest or during ADL. Doses of diuretics generally fluctuate at very high levels. More intensive management and surveillance startegies should be considered, which may in some cases reveal poor compliance that would compromise out comes with any therapy.	
**Level 5: Exertion intolerant – “Housebound”**	Variable urgency, depends on maintenance of nutrition, organ function and activity
Comfortable at rest and with ADL but unable to engage in any other activity, living predominantly within the house. Patients are comfortable at rest without any congestive symptoms, but may have underlying refractory elevated volume status, often with renal dysfunction.	
**Level 6: Exertion limited – “walking wounded”**	Variable, depends on maintenance of nutrition, organ function and activity level
Patients without evidence of fluid overload are comfortable at rest, and with activities of daily living and minor activities outside the home, but fatigues after the first few minutes of any meaningful activity. Attribution to cardiac limitation requires careful measurements of peak oxygen consumption, in some cases with hemodynamic monitoring to confirm severity of cardiac impairment.	
**Level 7: Advanced NYHA III– “too well”**	Transplant or circulatory support may not currently be indicated
Patients who are without current or recent episodes of unstable fluid balance, living comfortably with meaningful activities limited to mild physical exertion.	

### 5.1 Fourth Annual Report

The fourth annual report (Kirklin et al., 2012) summarizes and analyzes the first 5 years of patient and data collection. With the evolution of pump technology and the development of destination therapy, the percentage of implants with a primary strategy of bridge to transplant has decreased by 50% since 2006 (44% vs 23%). This reduction was accompanied by a slight increase of the bridge to candidacy strategy and the doubling of the proportion of implants in destination therapy (from 16% to 34% between 2006 and 2011). In the USA, the percentage of implants with an initial goal of bridge to recovery is still low. Among patients who received pumps as destination therapy, switching to continuous flow pumps was definitive by the end of 2010. The overall survival rate has increased steadily since 2006. In particular, the one-year survival was over 80% for the implants carried out between 2010 and 2011, while it stood at 60% for implants performed between 2006 and 2007.

### 5.2 Fifth Annual Report

The fifth annual report (Kirklin et al., 2013) summarizes and analyzes the first 6 years of patient and data collection. The current analysis includes more than 6000 patients and updated risk factors for continuous flow pumps. Among continuous flow pumps, the actuarial survival is 80% at 1 year and 70% at 2 years. The application of VADs as destination therapy has dramatically increased since the approval of a continuous-flow devices for DT in 2010. Currently, more than 40% of implants have been designated as DT. Among patients stratified to a DT designation, essentially 100% received a continuous-flow pump. Patients in INTERMACS Levels 1 and 2 have about a 5–8% decrease in 1-year survival compared with other INTERMACS levels. The interaction between age and INTERMACS Level demonstrates the reduced tolerance of the elderly when LVAD implantation occurs during acute cardiac decompensation. So the proportion of patients in progressive cardiac decompensation (Level 2) or cardiogenic shock (Level 1) at the time of implant has decreased from approximately 64% before 2011 to just less than 54% in 2012. Adverse event burden will play an important role in driving therapeutic choices for INTERMACS Levels 4 to 7. Severe renal dysfunction and progressive right ventricular dysfunction were associated with a major reduction in early survival; patients over 70 were not burdened by a significantly higher mortality compared to those over 50 but elderly patients had less tolerance for additional risk factors (Kirklin et al., 2013).

## 6. Conclusions

Recent technological advances improved outcomes and led to a greater utilization of VADs in the vast number of patients impaired with advanced HF and who are not candidates for transplants. In the future, thanks to the development of innovative solutions that enable the miniaturization of power systems and the total deliverability of the VAD, it is conceivable that, given the small number of donations (Hosenpud et al., 2000), the VAD will become a viable alternative to heart transplantation. Moreover, once the economic problems are overcome, the ventricular assist devices show the added advantage of ensuring availability independently of the waiting lists that instead weigh the option of transplantation. Currently, awaiting trials demonstrating unequivocally the effectiveness and efficiency of the new VAD, it is necessary to maintain a multidisciplinary approach involving close collaboration between the different professionals in order to improve all phases of therapy, considering the diversity of individual patients which determines their constant monitoring and modification.
